# Correlates of Overweight in Children and Adolescents Living at Different Altitudes: The Peruvian Health and Optimist Growth Study

**DOI:** 10.1155/2019/2631713

**Published:** 2019-08-01

**Authors:** Carla Santos, Alcibíades Bustamante, Donald Hedeker, Olga Vasconcelos, Rui Garganta, Peter T. Katzmarzyk, José Maia

**Affiliations:** ^1^CIFI^2^ D, Faculty of Sport, University of Porto, Porto, Portugal; ^2^Faculty of Physical Culture and Sports, National University of Education Enrique Guzmán y Valle, Lima, Peru; ^3^Department of Public Health Sciences, University of Chicago, Chicago, IL, USA; ^4^Pennington Biomedical Research Center, Louisiana State University, Baton Rouge, LA, USA

## Abstract

**Background and Aim:**

Overweight prevalence in children and adolescents shows great variability which is related to individual-level and environmental-level factors. The present study aimed to determine the prevalence of and factors associated with overweight in Peruvian children and adolescents living at different altitudes.

**Methods:**

8568 subjects, aged 6–16 y, from the sea level, Amazon, and high-altitude regions were sampled. Overweight was identified using BMI; biological maturation and physical fitness were measured; school characteristics were assessed via an objective audit.

**Results:**

Overweight prevalence decreased with age (28.3% at 6 y to 13.9% at 16 y); it was higher in girls (21.7%) than boys (19.8%) and was higher at the sea level (41.3%), compared with Amazon (18.8%) and high-altitude (6.3%) regions. Approximately 79% of the variance in overweight was explained by child-level characteristics. In Model 1, all child-level predictors were significant (*p* < 0.001); in Model 2, six out of nine added school-level predictors (number of students, existence of policies and practices for physical activity, multisports-roofed, duration of Physical Education classes, and extracurricular activities) were significant (*p* < 0.001); in Model 3, subjects living at high altitudes were less likely to be overweight than those living at the sea level.

**Conclusions:**

Child- and school-level variables played important roles in explaining overweight variation. This information should be taken into account when designing more efficient strategies to combat the overweight and obesity epidemic.

## 1. Introduction

The prevalence of childhood overweight and obesity has systematically increased in developed and developing countries over the last few decades [1]. It is considered a global epidemic and a public health crisis [2], given the adverse health consequences throughout the life course [3] as well as the related economic costs placing an undue strain on healthcare systems [4].

A systematic analysis [1] of available data from 1980 to 2013 showed that the combined prevalence of overweight and obesity has substantially increased in children and adolescents in developed countries from 16.9% to 23.8% in boys and from 16.2% to 22.6% in girls; in developing countries, this increase was from 8.1% to 12.9% in boys and from 8.4% to 13.4% in girls. This prevalence has also increased rapidly in low- and middle-income countries [5]. Recently, Yang et al. [6] reported overall prevalences of 13.4% for underweight and 21% for overweight (including obesity) among young adolescents in 58 low- and middle-income countries.

It has been suggested that increases in childhood overweight have been steeper in Latin American countries [7]. Currently, more than 20% (approximately 42.5 million) of Latin American children aged 0 to 19 yrs are overweight or obese [8], and Peru is no exception to the growing problem of childhood undernutrition and overnutrition [9]. For example, Pajuelo-Ramírez et al. [10] investigated the prevalence of overweight (including obesity) and chronic malnutrition in 6- to 9-year-old Peruvian children and showed values of 21.5% and 17.8%, respectively.

The development of overweight and obesity is multifaceted, involving biological and environmental factors [11]. Furthermore, girls tend to be more overweight and obese than boys [12]; overweight tends to be higher in older age-groups [13], and those ahead in their maturity status are more likely to be overweight and/or obese [14]. High levels of physical fitness have been shown to be an important contributing factor in maintaining healthier body weights during childhood [15].

It is likely that context-specific information is important to explain why children vary in their weight status [16], and this is apparently evident in Peru. Its territory spreads across three main areas (sea level, Amazon region, and high altitude) with their multifaceted geographical and socioeconomic features. Furthermore, Peru is facing different stages of nutritional [17] and epidemiological [18] transitions. Available information [19] shows differences in overweight and obesity prevalences in children living in different regions, with a very high prevalence in Lima (the capital city). Tarqui-Mamani et al. [20], using data from children aged 5–13 yrs, concluded that living in the urban areas and in Metropolitan Lima was related to a greater likelihood of being overweight than living in the Amazon region or at high altitudes. These results were consistent to those reported by Álvarez-Dongo et al. [21] in children and adolescents aged from 5 to 19 yrs of age.

Children and adolescents spend most of their awake time at school, and identifying school-related factors that may be associated to overweight/obesity is of importance. It has recently been suggested that the school context has links with child/adolescent overweight and obesity [22]. For example, O'Malley et al. [23] showed that USA schools with a high concentration of students from low socioeconomic status households have a greater chance of having higher proportions of obesity students. On the other hand, Pallan et al. [24] reported that, in United Kingdom schools, the only school-level variable associated with BMI z-score was time spent in Physical Education classes (minutes/week).

Very few studies have investigated the combined links of individual- and environmental-level correlates of childhood and adolescent overweight, and this information is not available in Peruvian youth. Furthermore, using a multilevel approach to tackle this complex issue could provide new insights for the development of intervention programs to prevent childhood overweight and obesity. Therefore, the present study has the following aims: (1) to determine the prevalence of overweight in Peruvian children and adolescents by age, sex, and geographical area of residence; (2) to examine the importance of biological characteristics (age, sex, maturation, and physical fitness total score), school-level contexts, and geographical area of residence in explaining variation in BMI categories.

## 2. Materials and Methods

### 2.1. Communities

Geographical heterogeneity in Peru is largely expressed in three areas with different altitudes: (1) sea level: in the coast, Barranco (58 m) is one of the 43 districts of Lima Province and is located on the shore of the Pacific Ocean; (2) Amazon region: in the jungle area, La Merced and San Ramon (751 m) are districts with geographical continuity and integrate the Chanchamayo province; (3) high altitude: Junín is localized on a plateau at 4107 m in the central part of the Andes mountain. Geographic, demographic, socioeconomic, and educational characteristics of these areas [25] are reported in Table 1.

### 2.2. Study Participants

The present cross-sectional sample comes from the *Peruvian Health and Optimist Growth Study* which, in summary, scrutinizes the main sources of variation in physical growth, motor development, and health in Peruvian children and adolescents living at different altitudes [26]. For this paper, we used a sample of 8568 children and adolescents (3914 boys and 4654 girls, aged 6–16 yrs) from 31 public schools located at the sea level, in the Amazon region, and at high altitude. The analytical sample was selected based on the availability of complete data of anthropometry, biological maturation, and three physical fitness tests (standing long jump, shuttle run, and 12 minute run test); furthermore, we did not consider subjects classified as underweight (*n*_total_ = 384; 4.5% ot the overall sample) because their frequency within each age category (2.2% at age 6, 3.6% at age 7, 6.2% at age 8, 3.7% at age 9, 3.8% at age 10, 4.5% at age 11, 4.1% at age 12, 5.7% at age 13, 4.3% at age 14, 2.6% at age 15, and 4.4% at age 16) and sex (from 3.5% in boys to 4.6% in girls) was very low.

Based on information obtained from the Ministry of Education [27] on the distribution of students enrolled in the 2009-10 academic year (date of data collection), it was decided to intentionally select 31 public education institutions located in urban areas, of which 13 belonged to kindergarten, 10 at the primary school, and 8 at the secondary school. A total of 38% of the total number of students enrolled in the study, that is, 4 out of every 10 students who regularly attended school. The sample only included native children and adolescents from their respective regions (i.e., no migrants were included), regardless of whether their parents or grandparents were migrants or not. We gathered information about their birth place and current place of residence or address residence and cross-checked this information with the participants' identity card. All assessments were carried out in Barranco between November and December (2009) and April and July (2010), in La Merced and San Ramon between May and August, and in Junín between September and October of 2009.

After initial political and educational contacts with local authorities in each city, formal permission was obtained from schools' governmental bodies to participate in the study. Informed consent was provided by parents/legal guardians. The Ethics Committee of the National University of Education Enrique Guzmán y Valle (UNE EGyV) as well as all school directors approved the project.  Table 2 shows the number of children and adolescents by age, sex, and geographical area of residence. The information about sample size and frequencies for BMI categories (normal weight and overweight) according to IOTF [29] and WHO [30] cutoff points, by age, sex, and geographical area of residence can be found in Supplementary Tables 1 and 2, respectively (please see these tables in the Supplementary Material for comprehensive data analysis).

### 2.3. Child-Level Correlates

#### 2.3.1. Anthropometry

All measurements were made according to standardized techniques [31]. Height and sitting height were measured with the head positioned to the Frankfurt plane to the nearest 0.1 cm with a portable stadiometer (Sanny, Model ES-2060); body weight was measured at the nearest 0.1 kg using a digital scale (Pesacon, Model IP68). Body mass index (BMI) was computed using the standard formula (weight (kg)/height (m)^2^), and subjects were classified as either normal weight or overweight (including obesity) according to the International Obesity Task Force (IOTF) [29] and World Health Organization (WHO) [30] cutoff points. A dummy coding was created, with normal weight coded as 0 (reference category) and overweight (including obesity) coded as 1.

#### 2.3.2. Biological Maturation

Biological maturation was assessed with the maturity offset [32] which estimates the distance, in decimal years, each subject is from the age at peak height velocity (PHV). Formulas are sex-specific and use chronological age, height, sitting height, and leg length. A positive (+) maturity offset indicates the number of estimated years a child is beyond PHV; a negative (−) maturity offset is the number of estimated years a child is before the PHV, whereas a zero value indicates that a child is experiencing his/her PHV.

#### 2.3.3. Physical Fitness

Three measures of physical fitness were used. Muscular strength (standing long jump), agility (shuttle run), and cardiorespiratory fitness (12 minute run) were obtained from two well-known test batteries, AAHPERD [33] and EUROFIT [34]. All tests were performed according to their standardized protocols: (1) Standing long jump test: each subject stood with feet apart behind the takeoff line and, without a preparatory run, were instructed to jump as far as possible. The maximum jumping distance was recorded. Two trials were given, and the best score was recorded as the maximum jumping distance in centimetres. (2) Shuttle run test: each subject performed five cycles (round-trip) at maximum speed between two lines separated by five meters. (3) 12 minute run test: in a previously delimited field, schoolchildren ran/walked the maximum possible distance in 12 minutes. All physical fitness test results were converted to z-scores using a grand mean centering, and a total physical fitness score (PF_z_) was obtained by summing all individual z-scores as suggested [35].

### 2.4. Sociogeographic Context

Geographical areas of residence were located at different altitudes: sea level (58 m), Amazon region (751 m), and high altitude (4107 m). A coding scheme was generated with two dummies: sea level, the reference category, was coded as 0 0, high altitude was 1 0, and Amazon region was 0 1.

### 2.5. School-Level Correlates

The description and inventory of characteristics associated with the school contexts were obtained via an objective school audit, using a modified and locally adapted version of the Healthy Eating and Physical Activity modules of the Healthy School Planner designed by the Joint Consortium for School Health [36]. This audit was led by the experienced research team members and maps various domains: (1) school characterization (school size: number of students was divided by 10 so as to have a more interpretable odds ratio; school setting: mixed (the reference category) and urban); (2) policies and practices for physical activity (specifically, the existence of policies and practices issued by the state, school board, or any other government agency to promote physical activity, health, and well-being of students and are organized by the school) (“no policies and practices” was the reference category); (3) physical structure of the school (playground area: “with obstacles” was the reference category and multisports-roofed: “yes” was the reference category); (4) Physical Education classes (frequency: “one” was the reference category and duration: “90 min” was the reference category); and (5) extracurricular activities (“yes” was the reference category).

### 2.6. Data Quality Control

To certify data quality, the following procedures were used: (1) training of all team members by experienced researchers in the correct use of the technical procedures of body measurement and physical fitness test protocols; (2) conducting a pilot study to assess the quality of data collection; (3) retesting of a random sample of 29 children and adolescents; and (4) estimating the interobserver technical error of the measurement for anthropometric data (height TEM = 0.2 cm; sitting height TEM = 0.1 cm; body mass TEM = 0.1 kg) and ANOVA-based intraclass correlation coefficient (ICC) for physical fitness tests (standing long jump: (ICC = 0.85), shuttle run (ICC = 0.81), and 12 minute run (ICC = 0.79)).

### 2.7. Statistical Analysis

Exploratory analyses, outlier identification, and normality checks were completed prior to further analyses. Differences in frequencies of overweight by age, sex, and geographical area of residence were computed using a chi-squared (*χ* ^2^) test. Differences in means and frequencies of child-level variables between geographical areas of residence were computed using analysis of variance (ANOVA), as well as *χ* ^2^ test, respectively. Additionally, the Tukey HSD test was used for multiple comparisons. All analyses were performed in SPSS software version 24.0.

Because our data had a hierarchical structure, i.e., children nested within schools, we used a two-level random intercept (multilevel) logistic regression model for BMI categories (normal weight versus overweight). A three-level structure was considered—subjects nested within schools which are nested within regions. However, with only three regions, it is not recommended to treat region as a level in a multilevel analysis [37]. Instead, dummy variables for region were included as predictors of BMI categories and were included in the fixed part of the model.

A series of nested models were fit to explain variation in BMI categories using the deviance statistic as a measurement of relative fit [38]; differences in deviances follow a *χ* ^2^ distribution with degrees of freedom equal to the difference in the number of estimated parameters from both models. It is expected that better fitting models have lower deviance values. Modelling was done in a sequential manner as generally advocated [37, 38]. Firstly, a null model (*M*_0_) was fit to the data to compute the intraclass correlation coefficient to estimate the variance accounted for by the random school effects in BMI categories. Secondly, Model 1 (*M*_1_) using child-level BMI predictors (age, sex, age-by-sex interaction, maturity offset, and PF_z_) was fit. Thirdly, in the Model 2 (*M*_2_), school-level covariates were added (number of students, school setting, policies and practices for physical activity, playground area, multisports-roofed, frequency and duration of Physical Education classes, and extracurricular activities). In the last model, Model 3 (*M*_3_), the predictor of geographical area of residence was added. Covariates were centered at their grand mean when necessary as is common in multilevel analysis [38]. Results are presented as odds ratios and their 95% confidence intervals (95% CI). The multilevel analyses was performed in SuperMix software [39], allowing a simultaneous estimation of all model parameters using maximum likelihood procedures. Statistical significance was set at 5%.

## 3. Results

Table 3 provides information about the prevalence of overweight (including obesity) among Peruvian children and adolescents, by age, sex, and geographical area of residence, based on IOTF and WHO cutoff points. Using a *χ* ^2^ test, significant differences (*p* < 0.05) in the prevalence of overweight were found by age (*χ* ^2^ = 125.53, *p* < 0.05), sex (*χ* ^2^ = 4.68, *p* < 0.05), and geographical area of residence (*χ* ^2^ = 782.51, *p* < 0.05). In the total sample, the prevalence of overweight was 20.8% (95% CI = 20.0–21.7). Further, the prevalence decreased with age (from 28.3%, 95% CI = 24.8–32.2 at 6 yrs to 13.9%, 95% CI = 10.4–17.4 at 16 yrs); was higher in girls (21.7%, 95% CI = 20.6–22.9) than in boys (19.8%, 95% CI = 18.5–21.0); and was higher among sea-level children and adolescents (41.3%, 95% CI = 39.1–43.7), followed by the Amazon region (18.8%, 95% CI = 17.6–19.9) and high altitude (6.3%, 95% CI = 5.3–7.3).

Table 4 provides descriptive statistics for the child-level variables. Statistically significant differences (*p* < 0.05) were found for all variables among subjects living at different altitudes. High-altitude subjects are relatively older than their peers from the other areas (*F* = 62.45, *p* < 0.05; 11.6 versus 11.2 and 10.8 yrs). On average, Amazon region subjects lag behind their peers in biological maturation (*F* = 75.19, *p* < 0.05), and sea-level children are more physically fit compared with their peers from high altitude (*F* = 1371.35, *p* < 0.05). The frequency of girls is higher than boys in the three geographical regions (sea level: girls 61.1%, boys 38.9%; Amazon region: girls 52.7%, boys 47.3%; high altitude: girls 51.4%, boys 48.6%) (*χ* ^2^ = 48.69, *p* < 0.05).

Descriptive statistics for the school-level variables are presented in Table 5. The number of students in the schools ranged from 96 to 1200; 83.3% of the schools were located in an urban region; 16.7% had policies, and 38.9% had practices for physical activity. Furthermore, 83.3% of schools had a recreation space without obstacles and 66.7% did not have multisports-roofed complexes. In 94.4% of schools, Physical Education classes were conducted once per week and 55.6% of them lasted >90 min. Finally, 77.8% of schools provided extracurricular activities for schoolchildren.

The results of the multilevel models, based on IOTF cutoff points, are presented in Table 6. The results are very similar (in magnitude and direction) for both criteria (for WHO results, please see Supplementary Table 3 in the Supplementary Material for comprehensive data analysis). The *M*_0_ indicated that school-level effects expressed by the intraclass correlation coefficient was 0.209, meaning that ∼21% of the total variance in BMI categories among subjects was at the school level and that ∼79% of the variance was explained by child-level characteristics, respectively.

Results from *M*_1_ (child-level predictors) showed that younger subjects (OR = 0.16, 95% CI = 0.14–0.18) as well as those ahead in their maturity status were more likely to be overweight (OR = 14.19, 95% CI = 12.00–16.78). The age-by-sex interaction was statistically significant (OR = 0.74; 95% CI = 0.70–0.78) revealing that boys tend to be less likely to be overweight than girls as they get older. Furthermore, being more physically fit (OR = 0.80; 95% CI = 0.76–0.84) protects subjects from being overweight. There was a significant reduction in deviance from *M*_0_ to *M*_1_ (from 7927.97 to 6524.56; *χ* ^2^_(5)_ = 1403.41, *p* < 0.001), showing the better fit of *M*_1_.

In *M*_2_, school-level predictors were added. Six out of nine predictors were statistically significant (*p* < 0.001). Children from schools with a higher number of students (OR = 1.02; 95% CI = 1.01–1.03) and without multisports-roofed (OR = 2.44; 95% CI = 1.53–3.89) were more likely to be overweight. Surprisingly, children and adolescents from schools with physical activity policies (OR = 5.09; 95% CI = 2.61–9.94) and practices (OR = 2.83; 95% CI = 1.83–4.38), with Physical Education classes with more than 90 minutes of duration (OR = 3.02; 95% CI = 1.84–4.96), as well as with extracurricular activities (OR = 0.59; 95% CI = 0.37–0.92), had a greater likelihood of being overweight. There was a reduction in deviance from *M*_1_ to *M*_2_ (from 6524.56 to 6480.93; *χ* ^2^_(9)_ = 43.63, *p* < 0.001), showing the better fit of *M*_2_.

In the last model (*M*_3_), geographical area of residence was added and results showed that subjects living at high altitude (OR = 0.08; 95% CI = 0.04–0.18) were less likely to be overweight than those living at the sea level. In this model, subject-level covariates remained similar as in *M*_1_ and *M*_2_, but the importance of some school-level covariates changed. Children and adolescents from schools with a lower number of students (OR = 0.97; 95% CI = 0.96–0.98), with playground areas with obstacles (OR = 0.09; 95% CI = 0.04–0.24), with multisports-roofed (OR = 0.40; 95% CI = 0.24–0.66), and with only one Physical Education class per week (OR = 0.03; 95% CI = 0.01–0.13), were more likely to be overweight. There was a reduction in deviance from *M*_2_ to *M*_3_ (from 6480.93 to 6453.66; *χ* ^2^_(2)_ = 27.27, *p* < 0.001), showing the better fit of *M*_3_.

## 4. Discussion

Since the last century, Peru has been experiencing epidemiological and nutritional transitions with unforeseen changes in the prevalences of overweight and obesity. Here, we reported a combined prevalence of 20.8% for overweight and obesity in children aged 6 to 16 yrs, as well as significantly lower prevalences at higher ages. Moreover, we found that children and adolescents living at the sea level had the highest overweight prevalence, followed by those from the Amazon region and high altitudes. Álvarez-Dongo et al.[21], using WHO cutoff points, reported a combined prevalence of 24.4% in children aged 5 to 9 yrs, decreasing to 14.2% in youth aged 10 to 19 yrs, which are smaller than what we reported using the same cutoff points within the age range of 6 to 9 yrs (31.6%) and 10 to 16 yrs (20.7%). In addition, they also reported a similar trend, with the highest prevalence of overweight and obesity found among children and adolescents living in the coastal regions. These geographical differences may be due to the differences in urbanization levels and lower poverty observed in coastal districts, which may lead to changes in lifestyles, namely, food habits and physical activity levels [40].

We also observed sex differences in the prevalence of childhood/adolescence overweight, favouring boys. Available systematic review [41] revealed similar trends within and between countries, emphasizing that in developing countries, these sex disparities are more exacerbated and are commonly observed before and during puberty. Several factors may contribute to this sex difference: firstly, boys and girls differ in body composition as well as in weight gain patterns [12]; secondly, boys tend to carry out heavy work and physical activities with greater energy expenditure, whereas girls are more responsible for household tasks, involving less energy expenditure [42].

A major finding of our study was that the main fraction of the total variance in BMI categories (∼79%) was explained by child-level characteristics. These results are congruent with those obtained in the International Study of Childhood Obesity, Lifestyle and the Environment (ISCOLE), where 90% of the variance in obesity was explained at the childhood level among 9- to 11-year-old children from 12 countries [43]. Further, ISCOLE demonstrated that 92% to 94% of the variance in BMI and waist circumference, respectively, was explained at the childhood level, with the remainder being explained at the school and site levels [44].

We also showed that younger children were more likely to be overweight, corroborating Cremm et al.'s [45] study with Brazilian children linking their results to the fact that younger children were more prone to consume healthier foods less frequently and engage in less moderate-to-vigorous physical activities or sports as compared to older children. Boys tended to be less likely to be overweight than girls as they get older (the age-by-sex interaction was statistically significant). There is a tendency for girls to surpass boys in their overweight trends, which are also linked to the fact that girls exhibit, on average, higher amounts of body fat and boys higher amounts of muscle mass [12]. These sex differences in body composition are evident from fetal life but emerge primarily during prepubertal and pubertal phases of life. Girls enter puberty earlier—they experience their midgrowth spurt and their adolescent spurt at earlier ages and undergo a more rapid pubertal transition, whereas boys have a substantially longer growth period [46]. Furthermore, the action of sex steroid hormones (estrogen) begins earlier in girls, preparing them for menarche, which results in a greater accumulation of body fat [47], predisposing them to a greater risk of being overweight.

We also showed that children and adolescents ahead in their maturity status were more likely to be overweight, which agrees with [48] data in Portuguese children. This is expected since previous research has shown that the timing of biological maturation further seems to contribute to greater adiposity development and early maturing youths usually are taller and heavier and have higher BMI than their later maturing peers [49]. As such, our results were expected since those closer to their PHV had higher BMI values and, consequently, had a greater likelihood of being overweight. However, to the best of our knowledge, we were not able to identify information about biological maturation and its associations with body weight in Peruvian children and adolescents to compare with our results. We were only able to find reports on girls [50] based on their age at menarche, showing that those living at the sea level had a higher BMI than those living at high altitude; their age at menarche occurred earlier which may also explain the higher prevalence of overweight previously reported among sea-level girls compared with their peers from the other regions.

Physical fitness is an important health marker [51], and there is emerging evidence showing that adequate physical fitness levels are associated with the prevention of overweight during childhood and adolescence, playing an important protective role in its rate of development [52]. Our results confirmed this link, showing that muscular strength, agility, and cardiorespiratory fitness have negative associations with overweight status. This trend was also found by McGavock et al. [53] in that low cardiorespiratory fitness and reductions in physical fitness over time were positively related to weight gain and increased risk of overweight in children 6–15 yrs old. Similarly, Stodden et al. [54] showed that the development of adequate levels of motor competence and health-related fitness may be a key component to prevent “unhealthy” weight gain in children and adolescents. Therefore, we emphasize the important role of physical fitness as a protective factor to reduce overweight/obesity when developing efficient intervention programs in children and adolescents.

Contextual information appears to be important in explaining why children vary in their weight status. Living at the sea level, particularly in the region of Barranco, was related to a greater likelihood of being overweight compared with high altitude, which may indicate that overweight is not yet a nationally distributed problem in this country [19]. One possible explanation for this discrepancy may be due to distinct lifestyle patterns in the three geographical areas. Barranco has a high density population of 10.132 inhabitants per km^2^, increasing public insecurity and the lack of public recreational infrastructures available to schoolchildren, contributing to the adoption of sedentary lifestyles. In addition, the existence of several fast food restaurants, convenience stores, and marketing of unhealthy food choices increases the likelihood of children acquiring unhealthy eating habits. Conversely, Chanchamayo and Junín have a lower population density (10.08 inhabitants per km^2^ and 12.22 inhabitnat per km^2^, respectively), and the majority of the schoolchildren participate in recreational activities, housework, and supporting agricultural activities in the field, showing higher levels of physical activity compared with their peers from the sea level. Additionally, the market penetration is less common and the main livelihoods are some tourism, mainly in the Amazon region, but predominantly stockbreeding and nondiversified agriculture, which limit the nutritional intake and, consequently, affect children and adolescent's health and weight status [42].

On the other hand, it has been suggested that lower obesity rates among highlanders as compared to lowlanders can also be explained by direct effects of living at high-altitude on human physiology (e.g., loss of appetite due to increased leptin concentrations [55] and increased thermogenesis due to cold temperatures that increase thyroid hormone [56] and catecholamine levels [57]). Thus, it is possible that living at high altitude has a weight-lowering effect *per se*, independent of various confounders factors, including multiple expressions of families and socioeconomic status as proposed by Woolcott et al. [58].

Additionally, lower obesity rates among high-altitude children and adolescents may also be considered in terms of the “reverse causality” theory. It suggests that overweight/obese individuals might tend to migrate to lower altitudes where the natural environment is friendlier in terms of performing their daily tasks/chores and activities and/or because they have easier access to healthcare services [58, 59]. However, our sample only included native children and adolescents from their respective regions (i.e., no migrants were identified).

It is widely accepted that schools are an important factor in understanding variability in childhood overweight [22]. Our results confirmed this, given that ∼21% of the total variance in BMI categories was attributed to the schools children attended. In previous studies [23, 24], lower values of variance (ranging from 2% to 5%) were found to explain school-level differences in the variability in the odds of a student being overweight. This discrepancy can be due to the higher dissimilarity observed across school environments in developing countries, such as in Peru, when compared to developed countries.

We found that children attending schools with a lower number of students were more likely to be overweight, whereas those attending schools with playground areas without obstacles and without multisports-roofed were less likely to be overweight. Because school size was defined according to the number of enrolled students, schools with more students apparently have more space and facilities for children to play during their recess time and have greater potential to increase children's physical activity levels [60], which could also promote positive effects on their weight status. Furthermore, it is possible that a playground without obstacles represents a greater free space for children to play, acting as a protective agent from overweight during childhood.

In an effort to fight childhood overweight and obesity, school-based healthy eating and physical activity programs have been implemented across the globe, providing opportunities to enhance children's future health and well-being [61]. Surprisingly, we found an inverse effect in our results probably because the effectiveness of these programs is not yet very well established. For example, in a systematic review of intervention studies, Campbell et al. [62] tracked only seven studies on prevention of childhood obesity: four revealed effective programs, whereas three did not. In fact, the lack of evidence exploring how policies and practices within the school system are associated with an individual student's risk of being overweight continues to represent an important gap in the available literature [61]. Finally, Peruvian students from schools with two Physical Education classes per week, with a maximum of 90 minutes' duration, were less likely to be overweight. In Peru, physical education classes are mandatory by government authorities, and all students are engaged in at least 1 class per week. As such, it is of no surprise that students who have 2 classes per week were less likely to be overweight.

This study is not without limitations. Firstly, the cross-sectional nature of the design does not allow a dynamic analysis of intraindividual changes and interindividual differences in overweight/obesity unfolding during childhood and adolescence. Yet, no study is apparently available in South America sampling hundreds/thousands of children and adolescents from different sociogeographic areas. Secondly, notwithstanding the size of our sample, it is not representative of the Peruvian population and care must be taken when trying to generalize our results. Thirdly, we were not able to obtain information about dietary habits and objective measures of physical activity. Fourthly, our results were not adjusted for the putative influence of family income and/or insurance status. Despite these shortcomings, the study has several merits: firstly, we sampled children aged 6 to 16 yrs which represent an important growth and developmental time window; secondly, we used standard methods and highly reliable data at the individual and school levels; thirdly, we relied on multilevel modelling to capture the complexity of the nested information—children within their schools; fourthly, the inclusion of three different geographical areas of residence permitted a more comprehensive interpretation of differences in childhood.

## 5. Conclusions

In summary, the present study showed that the prevalence of overweight in Peruvian children and adolescents decreased with age, was higher in girls, and was also higher at the sea level compared to the Amazon region and high altitude. Child-level variables (age, sex, maturation, PF_z_, and geographical area of residence), as well as school-level variables (number of students, policies and practices for physical activity, playground area, multisports-roofed, and frequency and duration of Physical Education classes), played important roles in explaining variations in childhood overweight. This information should be taken into account when designing more efficient strategies to combat the overweight and obesity epidemic.

## Figures and Tables

**Table 1:** Geographic, demographic, socioeconomic, and educational characteristics of the three geographical areas located in the central region of Peru.

Characteristics	Sea level (Barranco)	Amazon region (La Merced and San Ramon)	High altitude (Junín)
Geographic			
Area (km^2^)	3.33	5 315.07	883.8
Population density (people/km^2^)	10.132	10.08	12.22
Altitude (m)	58	751	4 107
Demographic			
Total population	34.378	53.590	10.796
Official language	Spanish	Spanish/Quechua	Spanish/Quechua
Ethnic composition	Mestizo/Blanco/Quechua/Afroperuano	Mestizo/Quechua/Blanco Afroperuano/Native Amazonian	Quechua/Mestizo/Blanco
Climate	Subtropical desert climate	Warm and rainy tropical climate	High mountain and mountain climate
Average annual temperature	18°C	24.6°C	7°C–12°C
Hours of sunlight and twilight	11 h25 min–12 h50 min	11 h29 min–12 h47 min	11 h28 min–12 h47 min
Socioeconomic			
Human Development Index (HDI)	0.72	0.52	0.44
Life expectancy at birth	79.08	65.49	69.14
Education (%)^1^	86.94	86.61	76.56
Literacy (%)^2^	99.35	90.97	89.84
Per capita family income	1440.6	785.1	512.7
Primary production	Trade/tourism	Agriculture/tourism	Stockbreeding/agriculture
Educational			
School age children	15.829	13.960	2.703
Public	8.881 (56.1%)	11.126 (79.7%)	2.601 (96.2%)
Private	6.948 (43.9%)	2.834 (20.3)	102 (3.8%)
Urban	15.829 (100%)	12.927 (92.6%)	2.678 (99.1%)
Rural	0 (0%)	1.033 (7.4%)	25 (0.9%)
Boys	7.118 (45.0%)	7.172 (51.4%)	1.359 (50.3%)
Girls	8.711 (55.0%)	6.788 (48.6%)	1.344 (49.7%)

^1^School age population. ^2^Children and adults of 15 years of age or more who know how to read and write.

**Table 2:** Children and adolescents sampled in the three geographical areas of the central region of Peru.

Age (yrs)^†^	Sea level (Barranco)		Amazon region (La Merced and San Ramon)		High altitude (Junín)		All areas	
	Girls	Boys	Girls	Boys	Girls	Boys	Girls	Boys
6	103	45	176	151	49	56	328	252
7	78	39	158	233	77	57	313	329
8	94	69	231	208	59	68	384	345
9	139	64	209	239	81	105	429	408
10	101	71	272	247	85	102	458	420
11	136	110	231	234	107	80	474	424
12	114	105	300	191	182	125	596	421
13	85	31	272	168	102	132	459	331
14	149	74	228	159	144	141	521	374
15	120	119	190	192	138	112	448	423
16	81	36	88	89	75	62	244	187
Total	1200	763	2355	2111	1099	1040	4654	3914

Data by age and sex. ^†^Age was used as decimals of a year, and children aged 5.5 to 6.49 yrs were designated as 6, children aged 6.50 to 7.49 yrs as 7, and so on [28].

**Table 3:** Prevalence of overweight (including obesity) (*n*% and 95% confidence intervals), based on IOTF and WHO cutoff points, by age, sex, and geographical area of residence.

Overweight (including obesity)				
	IOTF		WHO	
	*N*	% (95% CI)	*N*	% (95% CI)
Age				
6	164	28.3 (24.8–32.2)	199	34.3 (30.5–38.3)
7	158	24.6 (21.3–28.0)	196	30.5 (27.1–34.0)
8	174	23.9 (20.9–27.0)	215	29.5 (26.6–34.3)
9	220	26.3 (23.5–29.4)	267	31.9 (28.8–34.9)
10	163	18.6 (15.6–21.4)	204	23.2 (20.5–26.1)
11	240	26.7 (23.8–29.6)	275	30.6 (27.6–34.0)
12	207	20.4 (17.9–22.7)	252	24.8 (22.0–27.6)
13	138	17.5 (14.9–20.3)	162	20.5 (17.7–23.3)
14	139	15.5 (13.2–18.1)	149	16.6 (14.3–19.0)
15	120	13.8 (11.5–16.1)	127	14.6 (12.4–17.0)
16	60	13.9 (10.4–17.4)	63	14.6 (11.4–18.1)

Sex				
Girls	1009	21.7 (20.6–22.9)	1105	25.7 (24.3–27.0)
Boys	774	19.8 (18.5–21.0)	1004	23.7 (22.5–25.0)

Geographical area of residence				
Sea level	810	41.3 (39.1–43.7)	894	45.5 (42.5–47.7)
Amazon region	838	18.8 (17.6–19.9)	1025	23.0 (21.7–24.2)
High altitude	135	6.3 (5.3–7.3)	190	8.9 (7.8–10.1)

All	1783	20.8 (20.0–21.7)	2109	24.6 (23.7–25.5)

**Table 4:** Descriptive statistics for child-level variables.

Child-level variables	Sea level (S) (*n*=1963)	Amazon region (A) (*n*=4466)	High altitude (H) (*n*=2139)	*F*	*Post hoc* among areas
	Mean ± SD	Mean ± SD	Mean ± SD		
Biological characteristics					
Age (yrs)	11.20 ± 2.98	10.78 ± 2.87	11.61 ± 2.84	62.45^*∗*^	H > S > A
Maturity offset (yrs to PHV)	−1.27 ± 2.30	−1.89 ± 2.28	−1.30 ± 2.25	75.19^*∗*^	A < S; A < H
PF_z_	0.46 ± 1.76	0.52 ± 1.53	−1.46 ± 1.13	1371.35^*∗*^	H < S; H < A

	Frequencies *n* (%)			*χ* ^2^	

Sex					
Girls	1200 (61.1)	2355 (52.7)	1099 (51.4)	48.69^*∗*^	
Boys	763 (38.9)	2111 (47.3)	1040 (48.6)		

^*∗*^ *p* < 0.05; PF_z_ = total physical fitness score.

**Table 5:** Descriptive statistics for school-level variables.

School-level variables	Mean ± SD	Range
School characterization		
School size		
Number of students	453 ± 263	96–1200

	Frequencies *n* (%)	

School setting		
Mixed	3 (16.7)	
Urban	15 (83.3)	
Policies and practices for physical activity		
Policies and practices		
Only policies	3 (16.7)	
Only practices	7 (38.9)	
No	8 (44.4)	
Physical structure of the school		
Playground area		
With obstacles	3 (16.7)	
Without obstacles	15 (83.3)	
Multisports-roofed		
Yes	6 (33.3)	
No	12 (66.7)	
Physical Education classes		
Physical Education class frequency		
1 per week	17 (94.4)	
2 per week	1 (5.6)	
Physical Education class duration		
90 minutes	8 (44.4)	
>90 minutes	10 (55.6)	
Extracurricular activities		
Yes	14 (77.8)	
No	4 (22.2)	

**Table 6:** Multilevel modelling results: odds ratios (OR) and 95% confidence intervals (95% CI) for child- and school-level characteristics.

IOTF				
Parameters	Null model (*M*_0_)	Model 1 (*M*_1_)	Model 2 (*M*_2_)	Model 3 (*M*_3_)
Regression coefficients (fixed effects)				
Level-1: child level				
Intercept	0.18 (0.12–0.28)^*∗*^	5.71 (3.35–9.74)^*∗*^	1.23 (0.41–3.74)^ns^	33.48 (11.56–96.98)^*∗*^
Age		0.16 (0.14–0.18)^*∗*^	0.15 (0.14–0.18)^*∗*^	0.15 (0.14–0.17)^*∗*^
Sex (boys)^a^		2.71 (2.25–3.25)^*∗*^	2.74 (2.30–3.27)^*∗*^	2.94 (2.49–3.46)^*∗*^
Interaction (age-by-sex)		0.74 (0.70–0.78)^*∗*^	0.74 (0.70–0.78)^*∗*^	0.75 (0.72–0.79)^*∗*^
Maturity offset (yrs to PHV)		14.19 (12.00–16.78)^*∗*^	14.35 (12.14–16.97)^*∗*^	14.34 (12.12–16.96)^*∗*^
PF_z_		0.80 (0.76–0.84)^*∗*^	0.80 (0.76–0.84)^*∗*^	0.80 (0.76–0.83)^*∗*^
Geographical area of residence (high altitude)^¤^				0.08 (0.04–0.18)^*∗*^
Geographical area of residence (Amazon region)				1.50 (0.94–2.40)^ns^
Level-2: school level				
Number of students^†^			1.02 (1.01–1.03)^*∗*^	0.97 (0.96–0.98)^*∗*^
School setting (urban)^Ɵ^			0.58 (0.32–1.05)^ns^	1.19 (0.81–1.74)^ns^
Existence policies or/and practices for physical activity (policies)^∞^			5.09 (2.61–9.94)^*∗*^	5.64 (3.37–9.45)^*∗*^
Existence policies or/and practices for physical activity (practices)			2.83 (1.83–4.38)^*∗*^	1.40 (1.01–1.93)^*∗*^
Playground area (without obstacles)^£^			1.67 (0.62–4.51)^ns^	0.09 (0.04–0.24)^*∗*^
Multisports-roofed (no)^¥^			2.44 (1.53–3.89)^*∗*^	0.40 (0.24–0.66)^*∗*^
Frequency of physical education classes (two)^*α*^			1.87 (0.43–8.19)^ns^	0.03 (0.01–0.13)^*∗*^
Duration of physical education classes (>90 min)^§^			3.02 (1.84–4.96)^*∗*^	4.16 (3.04–5.69)^*∗*^
Extracurricular activities (no)^∆^			0.59 (0.37–0.92)^*∗*^	0.82 (0.60–1.12)^ns^
Variance components (random effects)				
Intercept	0.87 ± 0.30	1.07 ± 0.38	0.07 ± 0.03	0.00 ± 0.00
Model summary				
Deviance	7927.97	6524.56	6480.93	6453.66
Number of estimated parameters	2	7	16	18

^*∗*^ *p* < 0.001; ^ns^ = nonsignificant; ^a^girls are the reference; ^¤^sea level is the reference; ^†^divided by 10; ^Ɵ^mixed is the reference; ^∞^no policies and practices is the reference; ^£^with obstacles is the reference; ^¥^yes is the reference; ^*α*^one is the reference; ^§^90 min is the reference; ^∆^yes is the reference. PF_z_ = total physical fitness score.

## Data Availability

The datasets generated and analyzed during the current study are not publicly available due to privacy laws associated with children's data but are available with a data-sharing agreement as approved by the Ethics Committee of the National University of Education Enrique Guzmán y Valle, Lima, Peru.

## Supplementary Materials

Supplementary Materials Supplementary Table 1: sample size and frequencies (*n* (%)) for BMI categories (normal weight and overweight) according to IOTF cutoff points, by age, sex, and geographical area of residence. Supplementary Table 2: sample size and frequencies (*n* (%)) for BMI categories (normal weight and overweight) according to WHO cutoff points, by age, sex, and geographical area of residence. Supplementary Table 3: multilevel modelling results: odds ratios (OR) and 95% confidence intervals (95% CI) for child- and school-level characteristics. All these supplementary materials were referenced at appropriate sections in the manuscript.

## References

[1] Ng M., Fleming T., Robinson M. (2014). Global, regional, and national prevalence of overweight and obesity in children and adults during 1980–2013: a systematic analysis for the global burden of disease study 2013. *The Lancet*.

[2] Lobstein T., Baur L., Uauy R. (2004). Obesity in children and young people: a crisis in public health. *Obesity Reviews*.

[3] Guh D. P., Zhang W., Bansback N., Amarsi Z., Birmingham C. L., Anis A. H. (2009). The incidence of co-morbidities related to obesity and overweight: a systematic review and meta-analysis. *BMC Public Health*.

[4] Withrow D., Alter D. (2011). The economic burden of obesity worldwide: a systematic review of the direct costs of obesity. *Obesity Reviews*.

[5] Abarca-Gómez L., Abdeen Z. A., Hamid Z. A. (2017). Worldwide trends in body-mass index, underweight, overweight, and obesity from 1975 to 2016: a pooled analysis of 2416 population-based measurement studies in 128.9 million children, adolescents, and adults. *The Lancet*.

[6] Yang L., Bovet P., Ma C., Zhao M., Liang Y., Xi B. (2018). Prevalence of underweight and overweight among young adolescents aged 12–15 years in 58 low‐income and middle‐income countries. *Pediatric Obesity*.

[7] Filozof C., Gonzalez C., Sereday M., Mazza C., Braguinsky J. (2001). Obesity prevalence and trends in Latin‐American countries. *Obesity Reviews*.

[8] Rivera J. Á., de Cossío T. G., Pedraza L. S., Aburto T. C., Sánchez T. G., Martorell R. (2014). Childhood and adolescent overweight and obesity in Latin America: a systematic review. *The Lancet Diabetes & Endocrinology*.

[9] Pomeroy E., Stock J. T., Stanojevic S., Miranda J. J., Cole T. J., Wells J. C. K. (2014). Stunting, adiposity, and the individual-level “dual burden” among urban lowland and rural highland peruvian children. *American Journal of Human Biology*.

[10] Pajuelo-Ramírez J., Sánchez-Abanto J., Alvarez-Dongo D., Tarqui-Mamani C., Agüero-Zamora R. (2013). Overweight, obesity and chronic mal nutrition in 6 to 9 year-old children in Peru, 2009-2010. *Revista Peruana de Medicina Experimental y Salud Pública*.

[11] Narciso J., Silva A. J., Rodrigues V. (2019). Behavioral, contextual and biological factors associated with obesity during adolescence: a systematic review. *PLoS One*.

[12] Wells J. C. K. (2007). Sexual dimorphism of body composition. *Best Practice & Research Clinical Endocrinology & Metabolism*.

[13] Hanley A. J., Harris S. B., Gittelsohn J., Wolever T. M., Saksvig B., Zinman B. (2000). Overweight among children and adolescents in a native Canadian community: prevalence and associated factors. *American Journal of Clinical Nutrition*.

[14] Wang Y. (2002). Is obesity associated with early sexual maturation? A comparison of the association in American boys versus girls. *Pediatrics*.

[15] Kim J., Must A., Fitzmaurice G. M. (2005). Relationship of physical fitness to prevalence and incidence of overweight among schoolchildren. *Obesity Research*.

[16] Riedel C., von Kries R., Fenske N., Strauch K., Ness A. R., Beyerlein A. (2013). Interactions of genetic and environmental risk factors with respect to body fat mass in children: results from the ALSPAC study. *Obesity*.

[17] Mispireta M., Mispireta M. L., Rosas Á. M., Velásquez J. E., Lescano A. G., Lanata C. F. (2007). Transición nutricional en el Perú, 1991–2005. *Revista Peruana de Medicina Experimental y Salud Publica*.

[18] Huynen M., Vollebregt L., Martens P., Benavides B. M. (2005). The epidemiologic transition in Peru. *Revista Panamericana de Salud Pública*.

[19] Torres-Roman J., Urrunaga-Pastor D., Avilez J. L., Helguero-Santin L. M., Malaga G. (2018). Geographic differences in overweight and obesity prevalence in Peruvian children, 2010–2015. *BMC Public Health*.

[20] Tarqui-Mamani C., Alvarez-Dongo D., Espinoza-Oriundo P. (2018). Prevalencia y factores asociados al sobrepeso y obesidad en escolares peruanos del nivel primario. *Revista de Salud Pública*.

[21] Álvarez-Dongo D., Sánchez-Abanto J., Gómez-Guizado G., Tarqui-Mamani C. (2014). Sobrepeso y obesidad: prevalencia y determinantes sociales del exceso de peso en la población peruana (2009-2010). *Revista Peruana de Medicina Experimental y Salud*.

[22] Procter K. L., Rudolf M. C., Feltbower R. G. (2008). Measuring the school impact on child obesity. *Social Science & Medicine*.

[23] O’Malley P., Johnston L. D., Delva J., Bachman J. G., Schulenberg J. E. (2007). Variation in obesity among American secondary school students by school and school characteristics. *American Journal of Preventive Medicine*.

[24] Pallan M. J., Adab P., Sitch A. J., Aveyard P. (2014). Are school physical activity characteristics associated with weight status in primary school children? A multilevel cross-sectional analysis of routine surveillance data. *Archives of Disease in Childhood*.

[25] INEI (2017). Perfil sociodemográfico del Perú. https://www.inei.gob.pe/media/MenuRecursivo/publicaciones_digitales/Est/Lib1539/index.html.

[26] Bustamante A., Beunen G., Maia J. (2011). Como crecen y se desarrollan los niños y adolescentes en La Merced y San Ramón. *Alcances Para la Educación Física, el Deporte y la Salud*.

[27] ESCALE, 2010, http://escale.minedu.gob.pe/magnitudes

[28] Eveleth P., Tanner J. (1990). *Worldwide Variation in Human Growth*.

[29] Cole T. J. (2000). Establishing a standard definition for child overweight and obesity worldwide: International survey. *BMJ*.

[30] Onis M. (2007). Development of a WHO growth reference for school-aged children and adolescents. *Bulletin of the World Health Organization*.

[31] Lohman T., Roche A., Martorell R. (1988). *Anthropometric Standarization Reference Manual*.

[32] Mirwald R. L., Baxter-jones A. D. G., Bailey D. A., Beunen G. P. (2002). An assessment of maturity from anthropometric measurements. *Medicine & Science in Sports & Exercise*.

[33] AAHPERD (1980). *Health Related Physical Fitness: Test Manual*.

[34] EUROFIT (1993). *Handbook for the EUROFIT Tests of Physical Fitness*.

[35] Huang Y.-C., Malina R. M. (2007). BMI and health-related physical fitness in Taiwanese youth 9–18 years. *Medicine & Science in Sports & Exercise*.

[36] Joint Consortium for School Health (October 2018). Healthy school planner. http://www.healthyschoolplanner.uwaterloo.ca.

[37] Snijders T. A. B., Bosker R. J. (2012). *Multilevel Analysis: An Introduction to Basic and Advanced Multilevel Modeling*.

[38] Hox J. J., Moerbeek M., Van de Schoot R. (2018). *Multilevel Analysis: Techniques and Applications*.

[39] Hedeker D. (2008). *Supermix: Mixed Effects Models*.

[40] Hernández-Vásquez A., Bendezú-Quispe G., Díaz-Seijas D. (2016). Análisis espacial del sobrepeso y la obesidad infantil en el Perú, 2014. *Revista Peruana de Medicina Experimental y Salud Pública*.

[41] Kanter R., Caballero B. (2012). Global gender disparities in obesity: a review. *Advances in Nutrition*.

[42] Preston E. C., Ariana P., Penny M. E., Frost M., Plugge E. (2015). Prevalence of childhood overweight and obesity and associated factors in Peru. *Revista panamericana de salud pública*.

[43] Katzmarzyk P. T., Barreira T. V., Broyles S. T. (2015). Relationship between lifestyle behaviors and obesity in children ages 9–11: results from a 12-country study. *Obesity*.

[44] Katzmarzyk P. T., Broyles S. T., Chaput J.-P. (2018). Sources of variability in childhood obesity indicators and related behaviors. *International Journal of Obesity*.

[45] Cremm E., Leite F. H. M., Abreu D. S. C. D. (2012). Factors associated with overweight in children living in the neighbourhoods of an urban area of Brazil. *Public Health Nutrition*.

[46] Gasser T., Müller H.-G., Köhler W., Prader A., Largo R., Molinari L. (1985). An analysis of the mid-growth and adolescent spurts of height based on acceleration. *Annals of Human Biology*.

[47] Riggs B. L., Khosla S., Melton L. (2002). Sex steroids and the construction and conservation of the adult skeleton. *Endocrine Reviews*.

[48] Gomes T., Katzmarzyk P., dos Santos F., Souza M., Pereira S., Maia J. (2014). Overweight and obesity in Portuguese children: prevalence and correlates. *International Journal of Environmental Research and Public Health*.

[49] Ahmed M. L., Ong K. K., Dunger D. B. (2009). Childhood obesity and the timing of puberty. *Trends in Endocrinology & Metabolism*.

[50] Gonzales G., Villena A. (1996). Body mass index and age at menarche in Peruvian children living at high altitude and at sea level. *Human Biology*.

[51] Ortega F. B., Cadenas-Sanchez C., Lee D.-C., Ruiz J. R., Blair S. N., Sui X. (2018). Fitness and fatness as health markers through the lifespan. *Progress in Preventive Medicine*.

[52] Rodrigues L. P., Stodden D. F., Lopes V. P. (2016). Developmental pathways of change in fitness and motor competence are related to overweight and obesity status at the end of primary school. *Journal of Science and Medicine in Sport*.

[53] McGavock J. M., Torrance B. D., McGuire K. A., Wozny P. D., Lewanczuk R. Z. (2009). Cardiorespiratory fitness and the risk of overweight in youth: the healthy hearts longitudinal study of cardiometabolic health. *Obesity*.

[54] Stodden D. F., Goodway J. D., Langendorfer S. J. (2008). A developmental perspective on the role of motor skill competence in physical activity: an emergent relationship. *Quest*.

[55] Riepl R. L., Fischer R., Hautmann H. (2012). Influence of acute exposure to high altitude on basal and postprandial plasma levels of gastroenteropancreatic peptides. *PLoS One*.

[56] Basu R., Samet J. M. (2002). Relation between elevated ambient temperature and mortality: a review of the epidemiologic evidence. *Epidemiologic Reviews*.

[57] Antezana A. M., Richalet J. P., Noriega I., Galarza M., Antezana G. (1995). Hormonal changes in normal and polycythemic high-altitude natives. *Journal of Applied Physiology*.

[58] Woolcott O. O., Gutierrez C., Castillo O. A., Elashoff R. M., Stefanovski D., Bergman R. N. (2016). Inverse association between altitude and obesity: a prevalence study among andean and low-altitude adult individuals of Peru. *Obesity*.

[59] Woolcott O. O., Castillo O. A., Gutierrez C., Elashoff R. M., Stefanovski D., Bergman R. N. (2014). Inverse association between diabetes and altitude: a cross-sectional study in the adult population of the United States. *Obesity*.

[60] Cradock A. L., Melly S. J., Allen J. G., Morris J. S., Gortmaker S. L. (2007). Characteristics of school campuses and physical activity among youth. *American Journal of Preventive Medicine*.

[61] Veugelers P. J., Fitzgerald A. L. (2005). Effectiveness of school programs in preventing childhood obesity: a multilevel comparison. *American Journal of Public Health*.

[62] Campbell K., Waters E., O’Meara S., Summerbell C. (2001). Interventions for preventing obesity in childhood. A systematic review. *Obesity Reviews*.

