# Supplementary material for: Correlates of Overweight in Children and Adolescents Living at Different Altitudes: The Peruvian Health and Optimist Growth Study
Source: J Obes. 2019 Aug 1;2019:2631713. doi: 10.1155/2019/2631713 (PMC6701273; doi:10.1155/2019/2631713)
Supplement: Supplementary Materials — Supplementary Table 1: sample size and frequencies (n (%)) for BMI categories (normal weight and overweight) according to IOTF cutoff points, by age, sex, and geographical area of residence. Supplementary Table 2: sample size and frequencies (n (%)) for BMI categories (normal weight and overweight) according to WHO cutoff points, by age, sex, and geographical area of residence. Supplementary Table 3: multilevel modelling results: odds ratios (OR) and 95% confidence intervals (95% CI) for child- and school-level characteristics. All these supplementary materials were referenced at appropriate sections in the manuscript. [file 2631713.f1.docx]

**Supplementary table 1.** Sample size and frequencies [n (%)] for BMI categories (normal weight and overweight) according to IOTF cut-off points, by age, sex and geographical area of residence.

|  | **Sea-level**  (Barranco) | | | | **Amazon region**  (La Merced  and San Ramon) | | | | **High-altitude**  (Junín) | | | | **All areas** | | | | | | |
| --- | --- | --- | --- | --- | --- | --- | --- | --- | --- | --- | --- | --- | --- | --- | --- | --- | --- | --- | --- |
|  | Girls | Boys | Girls | Boys | Girls | Boys | Girls | Boys | Girls | Boys | Girls | Boys | Girls | Boys | Girls | | Boys | |  |
| Age (yrs) | Normalweight | | Overweight | | Normalweight | | Overweight | | Normalweight | | Overweight | | Normalweight | | Overweight | | |  |  |
| 6 | 62 (60.2) | 28 (62.2) | 41 (39.8) | 17 (37.8) | 118 (67.0) | 109 (72.2) | 58 (33.0) | 42 (27.8) | 46 (93.9) | 53 (94.6) | 3 (6.1) | 3 (5.4) | 226 (68.9) | 190 (75.4) | 102 (31.1) | 62 (24.6) | |  |  |
| 7 | 37 (47.4) | 13 (33.3) | 41 (52.6) | 26 (66.7) | 127 (80.4) | 185 (79.4) | 31 (19.6) | 48 (20.6) | 69 (89.6) | 53 (93.0) | 8 (10.4) | 4 (7.0) | 233 (74.4) | 251 (76.3) | 80 (25.6) | 78 (23.7) | |  |  |
| 8 | 52 (55.3) | 30 (43.5) | 42 (44.7) | 39 (56.5) | 191 (82.7) | 166 (79.8) | 40 (17.3) | 42 (20.2) | 59 (100) | 57 (83.8) | 0 (0.0) | 11 (16.2) | 302 (78.6) | 253 (73.3) | 82 (21.4) | 92 (26.7) | |  |  |
| 9 | 66 (47.5) | 28 (43.8) | 73 (52.5) | 36 (56.3) | 169 (80.9) | 188 (78.7) | 40 (19.1) | 51 (21.3) | 74 (91.4) | 92 (87.6) | 7 (8.6) | 13 (12.4) | 309 (72.0) | 308 (75.5) | 120 (28.0) | 100 (24.5) | |  |  |
| 10 | 61 (60.4) | 44 (62.0) | 40 (39.6) | 27 (38.0) | 227 (83.5) | 198 (80.2) | 45 (16.5) | 49 (19.8) | 85 (100) | 100 (98.0) | 0 (0.0) | 2 (2.0) | 373 (81.4) | 342 (81.4) | 85 (18.6) | 78 (18.6) | |  |  |
| 11 | 76 (55.9) | 54 (49.1) | 60 (44.1) | 56 (50.9) | 176 (76.2) | 178 (76.1) | 55 (23.8) | 56 (23.9) | 94 (87.9) | 80 (100) | 13 (12.1) | 0 (0.0) | 346 (73.0) | 312 (73.6) | 128 (27.0) | 112 (26.4) | |  |  |
| 12 | 56 (49.1) | 46 (43.8) | 58 (50.9) | 59 (56.2) | 247 (82.3) | 168 (88.0) | 53 (17.7) | 23 (12.0) | 174 (95.6) | 119 (95.2) | 8 (4.4) | 6 (4.8) | 477 (80.0) | 333 (79.1) | 119 (20.0) | 88 (20.9) | |  |  |
| 13 | 49 (57.6) | 21 (67.7) | 36 (42.4) | 10 (32.3) | 224 (82.4) | 140 (83.3) | 48 (17.6) | 28 (16.7) | 91 (89.2) | 127 (96.2) | 11 (10.8) | 5 (3.8) | 364 (79.3) | 288 (87.0) | 95 (20.7) | 43 (13.0) | |  |  |
| 14 | 106 (71.1) | 46 (62.2) | 43 (28.9) | 28 (37.8) | 188 (82.5) | 145 (91.2) | 40 (17.5) | 14 (8.8) | 137 (95.1) | 134 (95.0) | 7 (4.9) | 7 (5.0) | 431 (82.7) | 325 (86.9) | 90 (17.3) | 49 (13.1) | |  |  |
| 15 | 100 (83.3) | 87 (73.1) | 20 (16.7) | 32 (26.9) | 159 (83.7) | 171 (89.1) | 31 (16.3) | 21 (10.9) | 122 (88.4) | 112 (100) | 16 (11.6) | 0 (0.0) | 381 (85.0) | 370 (87.5) | 67 (15.0) | 53 (12.5) | |  |  |
| 16 | 64 (79.0) | 27 (75.0) | 17 (21.0) | 9 (25.0) | 75 (85.2) | 79 (88.8) | 13 (14.8) | 10 (11.2) | 64 (85.3) | 62 (100) | 11 (14.7) | 0 (0.0) | 203 (83.2) | 168 (89.8) | 41 (16.8) | 19 (10.2) | |  |  |
| **Total** | **729** | **424** | **471** | **339** | **1901** | **1727** | **454** | **384** | **1015** | **989** | **84** | **51** | **3645** | **3140** | **1009** | **774** | |  |  |

**Supplementary table 2.** Sample size and frequencies [n (%)] for BMI categories (normal weight and overweight) according to WHO cut-off points, by age, sex and geographical area of residence.

|  | **Sea-level**  (Barranco) | | | | **Amazon region**  (La Merced  and San Ramon) | | | | **High-altitude**  (Junín) | | | | **All areas** | | | | | |
| --- | --- | --- | --- | --- | --- | --- | --- | --- | --- | --- | --- | --- | --- | --- | --- | --- | --- | --- |
|  | Girls | Boys | Girls | Boys | Girls | Boys | Girls | Boys | Girls | Boys | Girls | Boys | Girls | Boys | Girls | | Boys | |
| Age (yrs) | Normalweight | | Overweight | | Normalweight | | Overweight | | Normalweight | | Overweight | | Normalweight | | Overweight | | |  |
| 6 | 60 (58.3) | 21 (46.7) | 43 (41.7) | 24 (53.3) | 113 (64.2) | 94 (62.3) | 63 (35.8) | 57 (37.7) | 44 (89.8) | 49 (87.5) | 5 (10.2) | 7 (12.5) | 217 (66.2) | 164 (65.1) | 111 (33.8) | 88 (34.9) | |  |
| 7 | 34 (43.6) | 12 (30.8) | 44 (56.4) | 27 (69.2) | 122 (77.2) | 162 (69.5) | 36 (22.8) | 71 (30.5) | 68 (88.3) | 48 (84.2) | 9 (11.7) | 9 (15.8) | 224 (71.6) | 222 (67.5) | 89 (28.4) | 107 (32.5) | |  |
| 8 | 46 (48.9) | 24 (34.8) | 48 (51.1) | 45 (65.2) | 190 (82.3) | 140 (67.3) | 41 (17.7) | 68 (32.7) | 59 (100) | 55 (80.9) | 0 (0.0) | 13 (19.1) | 295 (76.8) | 219 (63.5) | 89 (23.2) | 126 (36.5) | |  |
| 9 | 64 (46.0) | 23 (35.9) | 75 (54.0) | 41 (64.1) | 161 (77.0) | 167 (69.9) | 48 (23.0) | 72 (30.1) | 74 (91.4) | 81 (77.1) | 7 (8.6) | 24 (22.9) | 299 (69.7) | 271 (66.4) | 130 (30.3) | 137 (33.6) | |  |
| 10 | 56 (55.4) | 36 (50.7) | 45 (44.6) | 35 (49.3) | 224 (82.4) | 185 (74.9) | 48 (17.6) | 62 (25.1) | 79 (92.9) | 94 (92.2) | 6 (7.1) | 8 (7.8) | 359 (78.4) | 315 (75.0) | 99 (21.6) | 105 (25.0) | |  |
| 11 | 70 (51.5) | 47 (42.7) | 66 (48.5) | 63 (57.3) | 172 (74.5) | 164 (70.1) | 59 (25.5) | 70 (29.9) | 94 (87.9) | 76 (95.0) | 13 (12.1) | 4 (5.0) | 336 (70.9) | 287 (67.7) | 138 (29.1) | 137 (32.3) | |  |
| 12 | 52 (45.6) | 36 (34.3) | 62 (54.4) | 69 (65.7) | 235 (78.2) | 155 (81.2) | 65 (21.7) | 36 (18.8) | 170 (93.4) | 117 (93.6) | 12 (6.6) | 8 (6.4) | 457 (76.7) | 308 (73.2) | 139 (23.3) | 113 (26.8) | |  |
| 13 | 49 (57.6) | 16 (51.6) | 36 (42.4) | 15 (48.4) | 219 (80.5) | 130 (77.4) | 53 (19.5) | 38 (22.6) | 91 (89.2) | 123 (93.2) | 11 (10.8) | 9 (6.8) | 359 (78.2) | 269 (81.3) | 100 (21.8) | 62 (18.7) | |  |
| 14 | 103 (69.1) | 45 (60.8) | 46 (30.9) | 29 (39.2) | 184 (80.7) | 145 (91.2) | 44 (19.3) | 14 (8.8) | 137 (95.1) | 132 (93.6) | 7 (4.9) | 9 (6.4) | 424 (81.4) | 322 (86.1) | 97 (18.6) | 52 (13.9) | |  |
| 15 | 99 (82.5) | 87 (73.1) | 21 (17.5) | 32 (26.9) | 159 (83.7) | 166 (86.5) | 31 (16.3) | 26 (13.5) | 121 (87.7) | 112 (100) | 17 (12.3) | 0 (0.0) | 379 (84.6) | 365 (86.3) | 69 (15.4) | 58 (13.7) | |  |
| 16 | 62 (76.5) | 27 (75.0) | 19 (23.5) | 9 (25.0) | 75 (85.2) | 79 (88.8) | 13 (14.8) | 10 (11.2) | 63 (84.0) | 62 (100) | 12 (16.0) | 0 (0.0) | 200 (82.0) | 168 (89.8) | 44 (18.0) | 19 (10.2) | |  |
| **Total** | **695** | **374** | **505** | **389** | **1854** | **1587** | **501** | **524** | **1000** | **949** | **99** | **91** | **3549** | **2910** | **1105** | **1004** | |  |

**Supplementary Table 3.** Multilevel modelling results: odds ratios (OR) and 95% confidence intervals (95%CI) for child- and school-level characteristics

| **WHO** | | | | |
| --- | --- | --- | --- | --- |
| Parameters | Null model (M_0_) | Model 1 (M_1_) | Model 2 (M_2_) | Model 3 (M_3_) |
| *Regression coefficients (fixed effects)* | | | | |
| **Level-1 child-level** | | | | |
| Intercept | 0.23 (0.15-0.35)^*^ | 4.52 (2.77-7.39)^*^ | 0.95 (0.35-2.61)^ns^ | 22.61 (8.44-60.57)^*^ |
| Age |  | 0.19 (0.17-0.22)^*^ | 0.19 (0.17-0.21)^*^ | 0.19 (0.17-0.21)^*^ |
| Sex (boys)* |  | 3.31 (2.80-3.92)^*^ | 3.31 (2.81-3.89)^*^ | 3.51 (3.01-4.09)^*^ |
| Interaction (age-by-sex) |  | 0.74 (0.71-0.78)^*^ | 0.74 (0.71-0.78)^*^ | 0.75 (0.72-0.79)^*^ |
| Maturity offset (yrs to PHV) |  | 10.29 (8.85-11.97)^*^ | 10.44 (8.97-12.14)^*^ | 10.38 (8.92-12.08)^*^ |
| PF_z_ |  | 0.83 (0.79-0.86)^*^ | 0.83 (0.79-0.86)^*^ | 0.82 (0.79-0.86)^*^ |
| Geographical area of residence (High-altitude)^¤^ |  |  |  | 0.09 (0.04-0.18)^*^ |
| Geographical area of residence (Amazon region) |  |  |  | 1.09 (0.70-1.69)^ns^ |
| **Level-2 school-level** | | | | |
| Number of students† |  |  | 1.02 (1.01-1.02)^*^ | 0.98 (0.97-0.99)^*^ |
| School setting (urban)^Ɵ^ |  |  | 0.70(0.41-1.20)^ns^ | 1.27 (0.90-1.81)^ns^ |
| Existence policies or/and practices for physical activity (policies)^∞^ |  |  | 4.32 (2.35-7.93)^*^ | 3.64 (2.24-5.93)^*^ |
| Existence policies or/and practices for physical activity (practices) |  |  | 2.45 (1.65-3.64)^*^ | 1.22 (1.01-1.64)^*^ |
| Playground area (without obstacles)^£^ |  |  | 1.68 (0.68-4.16)^ns^ | 0.15 (0.06-0.36)^*^ |
| Multi-sports roofed (no)^¥^ |  |  | 2.22 (1.46-3.36)^*^ | 0.43 (0.27-0.68)^*^ |
| Frequency of physical education classes (two)^α^ |  |  | 1.94 (0.51-7.42)^ns^ | 0.07 (0.02-0.25)^*^ |
| Duration of physical education classes (>90 min)^§^ |  |  | 2.98 (1.89-4.69)^*^ | 3.60 (2.67-4.84)^*^ |
| Extracurricular activities (no)^∆^ |  |  | 0.61 (0.40-0.92)^*^ | 0.89 (0.67-1.19)^ns^ |
| *Variance components (random effects)* | | | | |
| Intercept | 0.80±0.28 | 0.90±0.32 | 0.05±0.03 | 0.00±0.00 |
| *Model summary* | | | | |
| Deviance | 8686.31 | 7385.03 | 7341.06 | 7314.74 |
| Number of estimated parameters | 2 | 7 | 16 | 18 |

^*^p<0.001; ^ns^= non-significant; *Girls are the reference; ^¤^Sea-level is the reference; †Divided by 10; ^Ɵ^Mixed is the reference; ^∞^No policies and practices is the reference; ^£^with obstacles is the reference; ^¥^Yes is the reference; ^α^One is the reference; ^§^90 min is the reference; ^∆^Yes is the reference; PF_z_ = total physical fitness score
